# 支气管镜下高频圈套器完全切除管内型错构瘤1例

**DOI:** 10.3779/j.issn.1009-3419.2011.11.13

**Published:** 2011-11-20

**Authors:** 继旺 王, 梅梅 李, 茂 黄, 文 刘, 栩 齐

**Affiliations:** 210029 南京，南京医科大学第一附属医院呼吸内科 Department of Respiratory Medicine, the First Affilliated Hospital, Nanjing Medical University, Nanjing 210029, China

管内型错构瘤是一种少见的良性肿瘤，传统的治疗方法为外科手术切除。而通过应用电圈套器对1例左主支气管管内型错构瘤进行了完全切除，效果良好，随访1年无复发，现报道如下。

## 1 资料与方法

### 1.1 临床资料

患者，男，69岁。因“咳嗽、咳痰2月余，气喘7天”于2010年2月1日入院。患者2个月前无明显诱因出现刺激性咳嗽、咳痰，痰白质稀，量不多，无痰中带血、发热及胸痛。曾在当地医院误诊为“慢性阻塞性肺病”而给予抗感染、平喘等治疗，症状无明显改善，仍反复阵发性剧烈咳嗽，咳时感胸闷、气喘。查血常规基本正常，胸部CT及三维成像示左主支气管占位（图 1A，图 1B），遂转我院并收住呼吸科。本次发病患者无大量脓痰，无发热及消瘦、盗汗，无吞咽困难、声音嘶哑，睡眠正常。患者40余年前曾患“肺结核”已治愈。否认“高血压、糖尿病”史，无外伤、手术史，吸烟40年，每日20支。患者入院时一般情况良好，神志清，精神可，发育正常。浅表淋巴结未触及肿大。气管居中，胸廓无畸形，双侧呼吸动度均等，叩诊清音，听诊左肺呼吸音稍低，未闻及啰音。患者入院后实验室检查示血尿常规、出凝血时间、肝肾功能、肿瘤标志物及心电图均正常，肺功能示：一秒用力呼气容积（forced expiratory volume in one second, FEV1）为1.84 L（占预计值66.7%），FEV1/用力肺活量（forced vital capacity, FVC）为57%，中度阻塞性通气功能障碍。入院后给予支气管镜检查，镜下可见左主支气管腔内一肿物，圆形，表面光滑，血管丰富（图 1C），活检钳钳取肿物时感觉十分光滑坚硬，后改为穿刺针对腔内肿物进行针吸活检，并应用带针活检钳探寻肿物游离度较大的一端为8点位置附近。肿物活检病理结果未发现肿瘤细胞，考虑良性病变可能。结合胸部CT考虑为带蒂的良性肿物，一周后在喉罩全麻下进行了支气管镜下介入切除。

**1:**
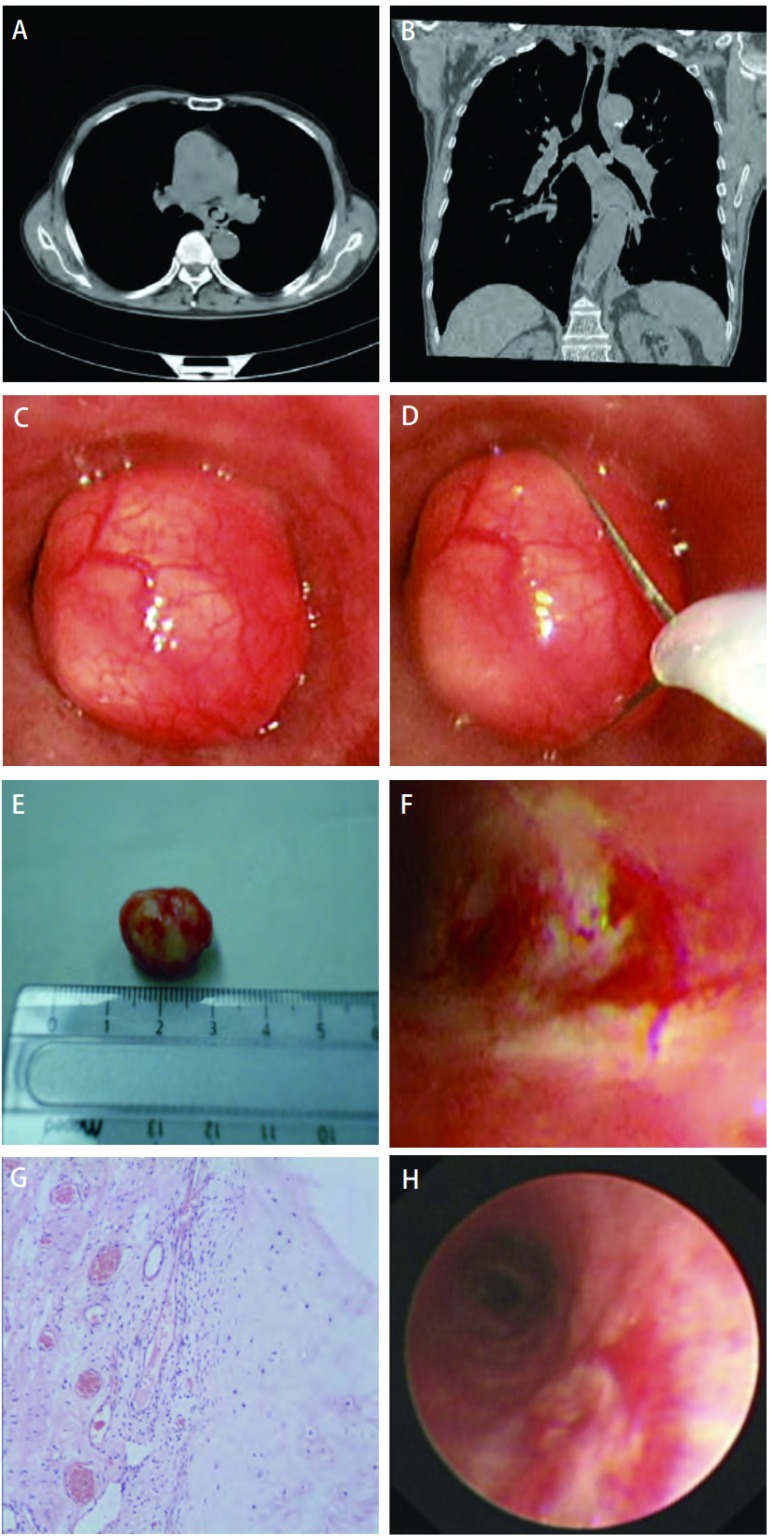
左主支气管腔内错构瘤及支气管镜下圈套器治疗效果。A：胸部CT显示左主支气管腔内一圆形肿物，密度不均匀; B：三维成像显示左主支气管一肿物，位于左主支气管管腔内，肿物内有高密度灶; C：电子支气管镜下见一圆形肿物，表面光滑，血管丰富; D：高频圈套器对左主支气管腔内带蒂肿物进行圈套切除; E：圈套器切除的肿物，大小约2.0 cm×1.5 cm; F：左主支气管腔内肿物被圈套器切除后腔内情况; G：组织病理显示：软骨组织及少许粘膜腺体（HE, ×100);H：一年后复查支气管镜显示：左主支气管腔内无明显异常。 The bronchoscopic effect of left main endobronchial hamartoma with theelectrosurgical snare. A: CT scan demonstrated a smooth surfaced round mass with uneven desity protruded from the left main bronchus; B: Three-dimensional imaging showed a mass from the left main bronchus with the high-density foci; C: Bronchoscopy showing a rounded mass with smooth surface, rich in blood vessels; D: The snare is useful for a polypoid lesion; E: The mass resected by the snare approximately 2.0 cm×1.5 cm; F: The view of the cavity of the left main bronchus with the mass resection by the snare; G: The cytologic examination view of the mass; H: The normal cavity in the left main bronchus one year later.

### 1.2 介入治疗方法

患者仰卧位，左下肢紧贴皮肤放置内衬盐水纱布的电极板。麻醉诱导前，静注阿托品0.5 mg、地塞米松10 mg以减少呼吸道分泌物及预防呼吸道粘膜水肿，在心电、脉氧及血压监测下，静脉推注咪唑安定2 mg、芬太尼50 μg、丙泊酚50 mg麻醉诱导后顺利插入4^#^喉罩（欧普乐牌，台湾旭邦工业有限公司），喉罩与呼吸机用三通连接管连接。丙泊酚、雷米芬太尼泵注维持麻醉。电子支气管镜（BF260型，日本Olympus公司）由三通连接管带有密封帽端口进入，到达病灶后将圈套器经活检孔送入左主支气管病灶处，当视野内看到绿色标记时，打开电切电凝电源开关，将高频电治疗仪（PSD30型，日本Olympus公司）电切的输出功率设定为35 W，电凝输出功率为35 W。首先将圈套器（SD-18c-1、SD-7c-1，日本Olympus公司）在肿物的游离端（即8点位置）环绕肿物（图 1D），助手逐渐拉紧收缩圈套器，应用电凝方式，足踏开关5 s-10 s，反复进行两次后顺利切除肿物，并用异物钳钳出（图 1E），左主支气管管腔完全通畅，未发现粘膜烧灼及穿孔（图 1F），肿物组织病理学检查结果为错构瘤（图 1G）。1个月后肺功能检查完全正常。1年后支气管镜检查示左主支气管病灶处无复发及其它异常（图 1H）。

## 2 讨论

肺错构瘤分为两类：较常见的肺内型和较少见的支气管管内型。研究^[1]^表明管内型错构瘤是一种少见的良性肿瘤，起源于管腔粘膜下的未分化间叶组织，除了有增生的粘膜腺体外，还可见到由原始间叶组织化生形成的骨、软骨、脂肪及平滑肌等。而本例切除的管内型错构瘤的主要成分为软骨和少许粘膜腺体组织（图 1G）。

胸部CT对支气管管内型错构瘤的诊断有一定的价值。由于管内型错构瘤往往有多种组织成分构成，其在胸部CT上多表现为密度不均匀，边界光滑清晰，局部富含脂肪组织或脂肪组织存在钙化灶。当CT显示肿块内脂肪组织或爆米花样钙化时有诊断价值^[2]^。

支气管镜检查对支气管管内型错构瘤有明确的诊断价值。支气管管内型错构瘤起源于气道管壁并向腔内生长，支气管镜下的特征为圆形肿块，边缘光滑，常常有蒂与管壁相连，无粘膜下浸润，病理活检能明确诊断。正是由于上述特征，肺功能检查表现为阻塞性通气功能障碍，在临床上常常表现为反复的咳嗽、咳痰，阻塞性肺炎或肺不张，咯血或呼吸困难，易误诊为慢性阻塞性肺病等。本例患者治疗前肺功能为中度阻塞性通气功能障碍，圈套器切除后肺功能完全恢复正常，而在临床上主要表现为反复的咳嗽、咳痰，后期伴有气喘等症状。

由于支气管管内型错构瘤为良性病变，内镜下的介入治疗为首选。对于带有蒂的管内型错构瘤，应用圈套器进行切割治疗，更有无可比拟的优势。传统的治疗手段为外科手术切除，创伤大、费用高。对于带蒂的管内型错构瘤，在应用圈套器进行切割前，明确蒂的位置非常关键。我们通常的做法是通过胸部CT及气道三维成像的情况来初步判断蒂的位置，同时在支气管镜下应用活检钳或带针活检钳钳住肿块进行上下移动，游离度小的部位可能是蒂的位置，然后在其对侧释放圈套器，若能深入下去，亦可判断游离端的位置，深入的圈套器向蒂的位置逐渐收紧进行套切，在套切时尽可能应用电凝或电凝切混合模式，以减少出血情况。

目前对于管内型错构瘤的介入治疗方法有多种，如硬质支气管镜下机械切除或联合激光、高频电灼等治疗亦取得良好效果，但由于硬质支气管镜介入治疗须在全麻下进行，且对于气道腔内远端病变的治疗成功率不高，对于有颈椎疾患、上下颌骨病变口腔不能张开及上颌面创伤时则属禁忌。本例患者肿块在左主支气管管腔内，亦可以在局麻下进行高频电切除，若选择在局麻下对气管及部分其它中心气道部位病变进行支气管镜介入治疗，由于反复电切割和电凝刺激及频繁咳嗽使血氧分压下降，存在窒息的风险，而且在清醒状态下实施治疗除了会引起恶心、呕吐、疼痛和不适外，还可能引起血压升高、心律失常等严重的应激反应，即使表面麻醉完善，术中取活检、出血、冲洗、吸引、激光、高频电和氩等离子进行烧灼、烟雾刺激等，患者也难免会出现呛咳、躁动、挣扎等状况，许多患者都感到十分紧张和恐惧，有时甚至终止和放弃治疗^[3]^。因此多选择在全身麻醉建立人工气道的情况下进行介入治疗。我们与患者沟通后采用了喉罩通气全麻下支气管镜下圈套器进行切除，在整个手术过程中患者各生命体征平稳，安全、简单，不失为一种良好的选择。

总之，随着呼吸介入技术的发展，对于某些气道病变，尤其是良性病变，已由传统的外科手术切除转为呼吸介入治疗，后者安全、简单、费用低，效果明显^[4]^。本例患者经呼吸介入方法进行了彻底治疗，不但患者花费少，更重要的是患者免除了手术之苦，治疗效果堪称完美，值得在临床上推广应用。
